# The impact of early empirical antibiotics treatment on clinical outcome of very preterm infants: a nationwide multicentre study in China

**DOI:** 10.1186/s13052-023-01414-x

**Published:** 2023-01-26

**Authors:** Yao Zhu, Qing Yang, Fan Wu, Jian Mao, Ling Liu, Rong Zhang, Wei Shen, Lixia Tang, Yanmei Chang, Xiuzhen Ye, Yinping Qiu, Li Ma, Rui Cheng, Hui Wu, Dongmei Chen, Zhi Zheng, Xiaomei Tong, Xinzhu Lin, on behalf of the Chinese Multicenter EUGR Collaborative Group, Qianxin Tian, Qiliang Cui, Ling Ren, Yuan Yuan, Bizhen Shi, Yumei Wang, Jinghui Zhang, Yan Zhu, Jingjing Zou, Yuhuai Li, Baoyin Zhao, Shuhua Liu, Ying Xu, Wenli Zhou, Zhiyong Liu, Jinzhi Gao, Jing Liu, Ling Chen, Cong Li, Chunyan Yang, Ping Xu, Yayu Zhang, Sile Hu, Hua Mei, Zuming Yang, Zongtai Feng, Sannan Wang, Eryan Meng, Lihong Shang, Falin Xu, Shaoping Ou, Rong Ju, Guinan Li, Juan Yi, Long Li, Yongqiao Liu, Zhe Zhang, Meigui Wu, Fei Bei, Ye Liu, Chun Deng, Huijie Yang, Ping Su, Shifeng Chen, Lingying Luo, Linlin Wang, Xiaohong Liu, Lihua Yan, Lijun Wang, Xiaokang Wang, Shuqun Yu, Qiaomian Zhu

**Affiliations:** 1 Department of Neonatology, Women and Children’s Hospital, School of Medicine, Xiamen University, Xiamen, 361003 Fujian China; 2 Xiamen Key Laboratory of Perinatal-Neonatal Infection, Xiamen, China; 3 Department of Neonatology, The Third Affiliated Hospital of Guangzhou Medical University, Guangzhou, China; 4 Department of Pediatrics, Shengjing Hospital of China Medical University, Shenyang, China; 5 Department of Neonatology, Guiyang Maternity and Child Health Hospital/Guiyang Children’s Hospital, Guiyang, China; 6 Department of Neonatology, Children’s Hospital of Fudan University, Shanghai, China; 7 Department of Pediatrics, Peking University Third Hospital, Beijing, 100074 China; 8 Department of Neonatology, Maternal and Children’s Hospital of Guangdong Province, Guangzhou, China; 9 Department of Neonatology, General Hospital of Ningxia Medical University, Yinchuan, China; 10 Department of Neonatology, Children’s Hospital of Hebei Province, Shijiazhuang, China; 11 Department of Neonatology, Children’s Hospital of Nanjing Medical University, Nanjing, China; 12 Department of Neonatology, The First Hospital of Jilin University, Changchun, China; 13 Department of Neonatology, Quanzhou Maternity and Children’s Hospital, Quanzhou, China

**Keywords:** Empirical antibiotics treatment, Very preterm infants, Weight growth velocity, Necrotizing enterocolitis

## Abstract

**Background:**

Infants with rule-out infections are responsible for the majority of empirical antibiotics treatment (EAT) in neonatal intensive care units (NICUs), particularly very preterm infants (VPIs). Antibiotic overuse has been linked to adverse outcomes. There is a paucity of data on the association between EAT and clinical outcomes (containing the nutritional outcomes) of VPIs without infection-related morbidities.

**Methods:**

Clinical data of VPIs admitted in 28 hospitals in 20 provinces of China from September 2019 to December 2020 were collected. EAT of VPIs was calculated as the number of days with initial usage in the first week after birth, and then categorized into 3 groups (antibiotic exposure: none, 1-4 days, and > 4 days). Clinical characteristics, nutritional status , and the short-term clinical outcomes among 3 groups were compared and analyzed.

**Results:**

In total, 1834 VPIs without infection-related morbidities in the first postnatal week were enrolled, including 152 cases (8.3%) without antibiotics, 374 cases (20.4%) with EAT ≤4 days and 1308 cases (71.3%) with EAT > 4 days. After adjusting for the confounding variables, longer duration of EAT was associated with decreased weight growth velocity and increased duration of reach of full enteral feeding in EAT > 4 days group (a*β*: -4.83, 95% *CI*: − 6.12 ~ − 3.53; a*β*: 2.77, 95% *CI*: 0.25 ~ 5.87, respectively) than those receiving no antibiotics. In addition, the risk of feeding intolerance (FI) in EAT > 4 days group was 4 times higher than that in non-antibiotic group (a*OR*: 4.14, 95%*CI*: 1.49 ~ 13.56) and 1.8 times higher than that in EAT ≤4 days group (a*OR*: 1.82, 95%*CI*: 1.08 ~ 3.17). EAT > 4 days was also a risk factor for greater than or equal to stage 2 necrotizing enterocolitis (NEC) than those who did not receive antibiotics (a*OR*: 7.68, 95%*CI*: 1.14 ~ 54.75) and those who received EAT ≤4 days antibiotics (a*OR*: 5.42, 95%*CI*: 1.94 ~ 14.80).

**Conclusions:**

The EAT rate among uninfected VPIs was high in Chinese NICUs. Prolonged antibiotic exposure was associated with decreased weight growth velocity, longer duration of reach of full enteral feeding, increased risk of feeding intolerance and NEC ≥ stage 2. Future stewardship interventions to reduce EAT use should be designed and implemented.

## Background

Given the immaturity of immune system, premature newborns are at a higher risk of infectious diseases such as sepsis, with atypical clinical manifestations, rapid disease progress, and high mortality [1, 2]. Therefore, prescription of empirical antibiotics treatment (EAT) is common among premature infants in neonatal intensive care unit (NICU). Over 75% of very low birth weight infants (VLBWI) and over 90% of extremely preterm infants (< 28 weeks of gestation) receive empirical antibiotics within the first postnatal week due to risk of early-onset sepsis (EOS) [3, 4]. Encouragingly, a large reduction in antibiotic use among preterm infants is observed and believing this is the result of increasing national focus on antibiotic overuse and misuse over the last decade. There was a marked reduction in the proportion of early antibiotics exposure declining from 82% in 2009 to 66% in 2018 across Norway including 4932 infants with a gestational age < 32 weeks, owing to the implementation of national antimicrobial stewardship [5]. Nevertheless, inappropriate antibiotic use and prolonged antibiotic durations still existed and became a big concern in premature newborns with rule-out infections [6]. Cohort and retrospective studies demonstrated that early EAT in very premature infants (VPIs) can increase the risk of poor prognosis, such as bronchopulmonary dysplasia (BPD), late-onset sepsis (LOS), neonatal necrotizing enterocolitis (NEC) and even death [6, 7]. It can also increase the risk of childhood asthma and obesity [8, 9]. Additionally, there was a dose-effect relationship (positive correlation) between duration of EAT and severity of those diseases [6, 7]. However, the most concerning finding was that 92% of EAT courses for rule-out sepsis were longer than 3 days with a median duration of 8 days [10]. Currently, clinicians do not pay enough attention to the harmfulness of EAT in preterm infants with rule-out infections, and the effect of in-hospital EAT on the clinical outcomes of VPIs without infection-related morbidities has not been sufficiently investigated. Thus, the aims of this retrospective cohort study were to compare the short-term outcomes of VPIs without infection-related morbidities who received different durations of empirical antibiotic exposure with initial usage in the first postnatal week, particularly on the nutritional outcomes.

## Methods

### Study population and design

The data of this study came from a prospective multicenter study that involved the influencing factors of extrauterine growth retardation (EUGR) in VPIs from different regions of China (The clinical trial registration: chictr.org.cn; Number: ChiCTR1900023418; Date of first registration: 26/05/2019). The study was approved by the Ethics Committee of the Women and Children’s Hospital, School of Medicine, Xiamen University (No. KY-2019-016). In the study, clinical data of VPIs in NICU were collected from 28 tertiary first-class hospitals in 20 provinces of China from September 2019 to December 2020.

#### Inclusion criteria

Infants born with gestational age (GA) < 32 week and admitted to the participating NICUs within 24 hours after birth.

#### Exclusion criteria

(I) Infants with congenital malformations and metabolic diseases; (II) infants who died, whose treatment was interrupted and led to an automatic discharge due to parental wishes based on financial constraints; (III) infants with incomplete medical record information. Based on this, infants who developed culture-proven sepsis, high-risk category of early-onset sepsis (EOS) with RCL score 3 (Table 1) [11], clinically diagnosed LOS, NEC, or other confirmed infectious diseases (including pneumonia, urinary tract infection, intracranial infection, or skin infection) during the first postnatal week were excluded.

**Table 1:** Risk classification of early-onset sepsis using an easy-to-use scoring system based on risk factors, clinical symptoms, and laboratory findings (RCL score)

Risk factors (R)	Clinical symptoms (C)	Laboratory findings (L)
1.Mother Group B streptococcus positive	1. Respiratory distress or apnoea	1. White blood cells < 5 × 10^9^/ L
2.Maternal chorioamnionitis (fever > 38.5 °C, fetal tachycardia)	2. Tachycardia or bradycardia	2. C-reactive protein > 10 mg/L
	3.Arterial hypotension and/or poor perfusion	
3.Premature rupture membranes > 18 hours	4. Hypothermia or hyperthermia	
4.Gestational age < 37 weeks	5. Seizure, floppy infant, irritability, or lethargy	
	6.Vomiting or feeding intolerance or ileus	

One point was given if one or more of the risk factors (R), the clinical symptoms (C) or the laboratory findings (L) were positive; The minimum score was 0, and the maximum score was 3 points

Low-risk category: neonates with one or no abnormal finding of the three areas of RCL, total score 0 or 1

Medium-risk category: neonates with abnormal findings in two of the three areas of RCL, total score 2

High-risk category: neonates with risk factors, clinical signs, and abnormal routine laboratory values, total score 3

### Study data set

This was a retrospective multicenter cohort study. VPIs who met the inclusion criteria were divided into three groups according to the duration of empirical use of antibiotics: non-antibiotic group, EAT ≤4 days group, EAT > 4 days group. The demographic and clinical data were collected including perinatal information (e.g. GA, BW, the use of antenatal steroids, birth mode, Apgar score at 5 minutes, maternal complications during pregnancy), and primary clinical outcome occurring after the first postnatal week, such as greater than or equal to stage 2 NEC (NEC ≥ stage 2), hospital-acquired infections (HAI), hemodynamically significant patent ductus arteriosus (hsPDA), severe intraventricular hemorrhage (IVH) (grade 3 or 4), periventricular leukomalacia (PVL), moderate and severe BPD, parenteral nutrition associated cholestasis (PNAC), and stage 3 through 5 retinopathy of prematurity (ROP) in either eye. We also collected nutrition related data, including breast feeding, use of breast milk fortifier, cumulative fasting time, and duration of parenteral nutrition.

### Study definitions

#### Early empirical antibiotics treatment (EAT)

With initial usage in the first week after birth, suspected bacterial infection among the VPIs was managed with empirical antibiotics depending on the suspected infection site, perinatal condition, treatment response, and based on local bacterial drug resistance surveillance data but did not have culture-proven infection.

#### Definitions related to EOS and LOS

(I) Risk classification of EOS (Suspected EOS): The probability of EOS was assessed using an easy-to-use scoring system based on risk factors (R), clinical symptoms (C), and laboratory findings (L) (Table 1) [11]. One point was given if one or more of the three areas of RCL were positive. The minimum score was 0, and the maximum score was 3 points. The neonates were stratified into three risk categories of EOS according to RCL score. Low-risk category defined as neonates with one or no abnormal finding of the three areas of RCL, total score 0 or 1; Medium-risk category defined as neonates with abnormal findings in two of the three areas of RCL, total score 2; High-risk category defined as neonates with risk factors, clinical signs, and abnormal routine laboratory values, total score 3.

(II) Culture-proven EOS: neonates 1-3 days with positive blood/CSF culture, and total RCL score ≥ 1;

(III) Clinically diagnosed late-onset sepsis (LOS): neonates aged > 3 days with clinical symptoms, and conform to any of the following conditions (1) two or more of abnormal routine laboratory values (white blood cells, immature/total neutrophil, C-reactive protein, platelets, and procalcitonin); (2) CSF examination indicates purulent meningitis; (3) bacterial DNA is detected in blood samples [12].

(IV) Culture-proven LOS: neonates aged > 3 days with positive blood/CSF culture and clinical symptoms.

#### Definitions related to nutrition management

(I) Days of reach of full enteral feeding were defined as the duration of oral feeding reaching 150 ml/ (kg.d); (II) Age of oral calorie attainment was defined as age of oral total calorie reaching 110 kcal/ (kg.d); (III) Weight growth velocity (GV) (after regaining birth weight) was calculated using an exponential model [13]; (IV) Breast-feeding was defined as the amount of breastfeeding accounting for more than 50% of the total enteral feeding during hospitalization; (V) EUGR was defined as postnatal weight below the 10th percentile of the expected growth for the postmenstrual age (PMA) at the time of discharge or 36 weeks PMA, and was evaluated with the 2013 Fenton Preterm Growth Chart [14]; (VI) The diagnostic criteria of feeding intolerance (FI) were in line with the Clinical guidelines for the diagnosis and treatment of FI in preterm infants (2020) [15].

#### Definitions related to primary clinical outcome

(I) Moderate to severe BPD was defined as requirement of oxygen therapy, positive pressure ventilation, or mechanical ventilation at the corrected GA of 36 weeks or at discharge (whichever comes first) according to National Institutes of Health (NIH) 2001 definition [16]; (II) There is no consensus on the definition of a hsPDA, in our study, hsPDA was defined as patent ductus arteriosus (PDA) catheter diameter > 1.5 mm, left atrium-to-aortic root (LA/Ao) ratio ≥ 1.4 accompanied by one of the following clinical manifestations: heart murmur, tachycardia (sustained ≥160 beats/min), increased respiration, increased pulse pressure (> 25 mmHg), hypotension, flushing, or cardiac dilation [17]; (III) Greater than or equal to stage 2 NEC based on the Bell criteria [18]; (IV) Hospital-acquired infections (HAI) was defined as confirmed infectious diseases including LOS, meningitis, pneumonia, or urinary tract infection with/without positive culture results after the first postnatal week (all occurring after EAT use); (V) Severe IVH (grade 3 or 4) based on the Papile criteria [19]; (VI) ROP staging was performed in agreement with international classification [20], and ROP requiring intervention was defined as ROP requiring intravitreal drug injection, laser therapy, or surgery; (VII) The diagnoses of arenteral nutrition associated cholestasis (PNAC), and PVL established by referring to Practical Neonatology (5th edition) [21].

### Statistics analysis

The counting data rate (%) indicated that comparison between groups was performed using χ2 test or *Fisher* exact probability method. *Kolmogorov-Smirnov* test was used to evaluate whether the measurement data conformed to the normal distribution. The measurement data of the normal distribution were expressed by $\overline{x}\pm s$, and the two independent samples *t*-test was for comparison between groups, while the measurement data of non-normal distribution was expressed by *M (Q1, Q3)*. The ranks sum test was used for group comparisons. Multivariate analysis was performed using binary logistic regression analysis and linear regression analysis. All statistical analyses were conducted using a software program (SPSS, version 26.0; IBM, Armonk, NY, USA), with statistical significance evaluated using 2-sided *P* values at the 5% testing level.

## Results

### General information

**Fig. 1:**
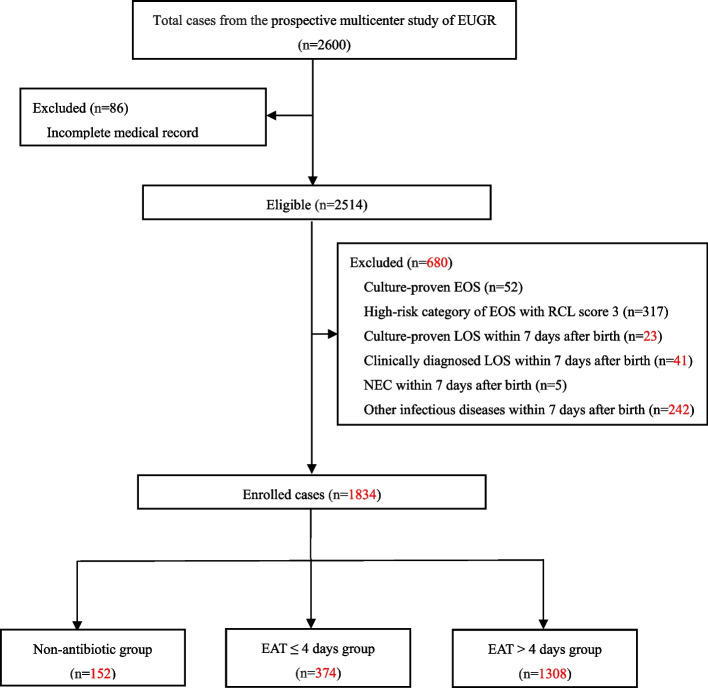
Subject enrollment and selection flow chart

**Fig. 2:**
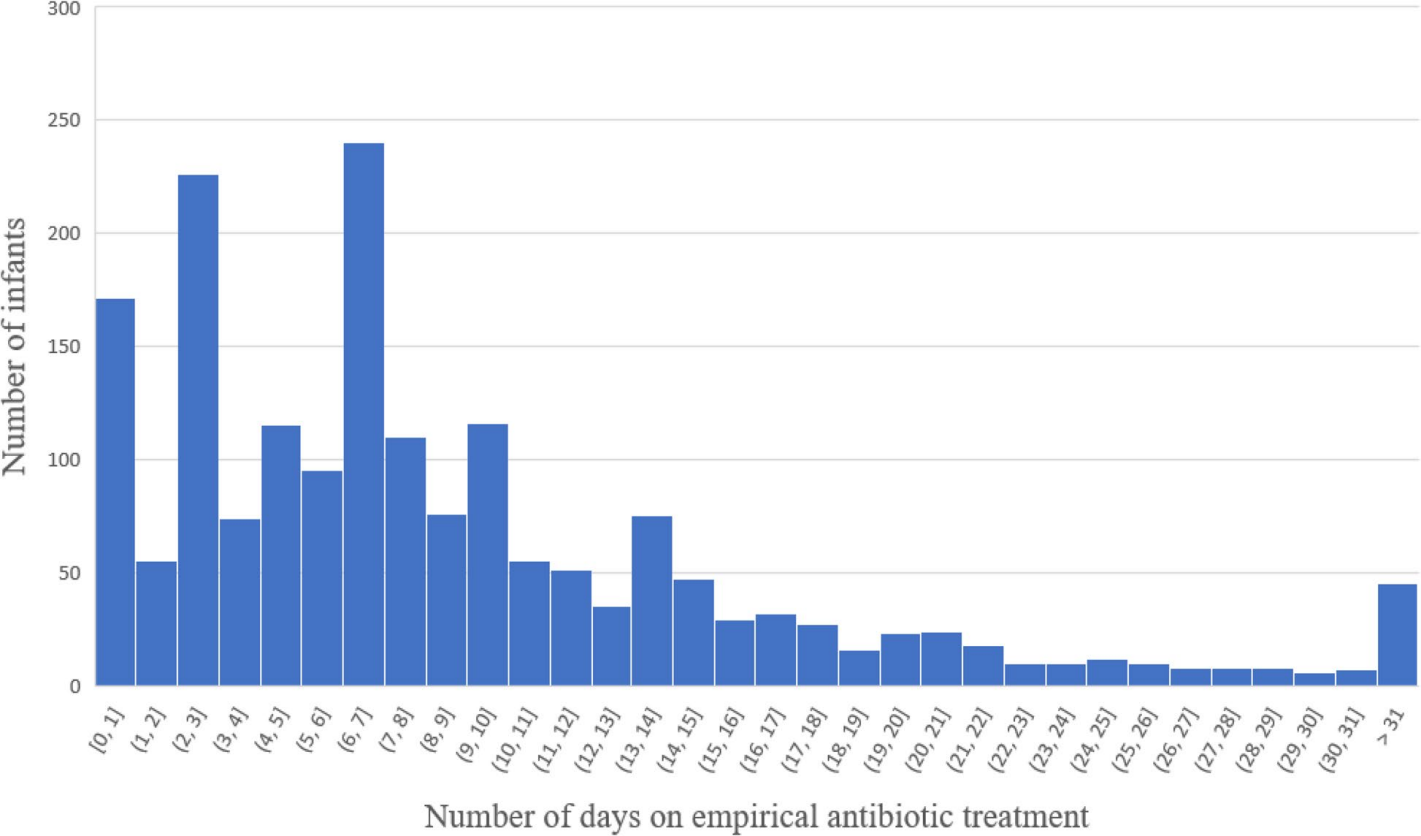
Numbers of study infants according to duration of empirical antibiotic treatment

A total of 2600 cases who met the inclusion criteria were enrolled through a prospective multicenter study of VPIs-EUGR from September 2019 to December 2020. Of these, 766 infants were excluded from analysis, including 86 cases with incomplete medical record data, 369 cases with EOS [317 cases with high-risk category of EOS (RCL score 3), 52 cases with culture-proven EOS], 64 cases with LOS during the first postnatal week (23 cases with culture-proven LOS, 41 cases with clinically diagnosed LOS,), 5 cases of early NEC (within 7 days after birth) and 242 cases of other infectious diseases within 7 days after birth such as pneumonia, urinary tract infection, intracranial infection, or skin infection. Therefore, the remaining 1834 uninfected infants during the first postnatal week were enrolled in the analysis. Among them, there were 152 cases (8.3%) who not treated with antibiotics, 374 cases (20.4%) with EAT≤4 days and 1308 cases (71.3%) with EAT > 4 days (Fig. 1). The total rate of EAT was 91.7% (1682/1834). Among 1682 infants who received EAT, 1501(89.2%) had treatment initiated at Day 0, and a total of 2622 courses were prescribed. There were 1095(65.1%), 294 (17.5%), and 293 (17.4%) infants who received 1, 2, and more than 2 courses of antibiotics, respectively. The rate of EAT in VPIs without infection-related morbidities among the hospitals involved in this study fluctuated between 41.3 and 97.6%. The median time of EAT usage during hospitalization was 9 days; 61.4% of them were 1-10 days (Fig. 2). There were 946 (56.2%), 687 (40.8%), 436 (25.9%), and 149 (8.9%) infants who received a ampicillin, third-generation cephalosporin, piperacillin tazobactam, and carbapenem, respectively.

### Comparison of perinatal and clinical data

Results showed that infants with lower GA and BW, more postnatal corticosteroid use, longer duration of mechanical ventilation and hospital stay, had higher level of empirical antibiotics usage (all *P* < 0.001). More empirical antibiotics usage was found in male infants and infants with Apgar score ≤ 7 at 5 min (*P* = 0.016 and 0.012, respectively). There was no significant difference in the use of empirical antibiotics for multiple births, cesarean section, small for gestational age (SGA), premature rupture of membranes (PROM) > 18 hours, completed antenatal steroids, gestational diabetes mellitus (GDM), and gestational hypertension among the three groups (all *P* > 0.05) (Table 2).

**Table 2:** Comparison of perinatal and clinical data of VPIs without infection-related morbidities

Variable	non-antibiotic	EAT ≤4 days	EAT > 4 days	*Z/χ*^*2*^	*P*
	(*n* = 152)	(*n* = 374)	(*n* = 1308)		
GA, [*M*(*Q1,Q3*)], weeks	31.2 (30.3,31.7)	30.6 (29.8,31.6)	30.2 (29.1,31.1)^a,b^	52.165	< 0.001
BW, [*M*(*Q1,Q3*)], grams	1510 (1300,1720)	1460 (1220,1630)	1340 (1160,1560)^a,b^	31.253	< 0.001
Multiple gestation, *n*(%)	42 (27.6)	111 (29.7)	455 (34.8)	5.701	0.058
Male gender, *n*(%)	67 (44.1)	216 (57.8) ^a^	718 (54.9)^a^	8.333	0.016
Cesarean section, *n*(%)	94 (61.8)	245 (65.5)	831 (63.5)	0.765	0.682
SGA, *n*(%)	18 (11.8)	44 (11.8)	143 (10.9)	0.276	0.871
Apgar score ≤ 7 at 5 min, *n*(%)	15 (9.9)	19 (5.1)	131 (10.0)^b^	8.805	0.012
PROM > 18 hours, *n*(%)	13 (8.6)	53 (14.2)	207 (15.8)	5.875	0.053
Completed antenatal steroids^c^, *n*(%)	75 (49.3)	174 (46.5)	607 (46.4)	0.476	0.788
Gestational diabetes mellitus, *n*(%)	32 (21.1)	59 (15.8)	235 (18.0)	2.173	0.337
Gestational hypertension, *n*(%)	27 (17.8)	75 (20.1)	221 (16.9)	2.001	0.368
Postnatal corticosteroid use, *n*(%)	3 (2.0)	14 (3.7)	285 (21.8)^a,b^	99.163	<0.001
Duration of mechanical ventilationd, [*M*(*Q1*,*Q3*)], days	7.4 (2.9,15.0)	8.1 (3.0,19.7)	16.0 (7.0,30.0)^a,b^	89.815	<0.001
Length of hospital stay, [*M*(*Q1*,*Q3*)], days	33.0 (24.0,42.0)	36.0 (29.0,46.0)^a^	44.0 (34.0,57.0)^a,b^	111.334	<0.001

^a^ Significantly different between the non-antibiotic group and EAT group

^b^ Significantly different between the EAT ≤4 days group and EAT > 4 days group

^c^ Intramuscular steroids cycle in two doses of 12 mg over a 24-h period

^d^ Mechanical ventilation includes invasive mechanical ventilation and non-invasive mechanical ventilation

*GA* gestational age, *BW* birth weight, *SGA* small for gestational age, *PROM* premature rupture of membranes

### Comparison of nutritional outcomes

After adjusting for confounding variables, linear regression analysis indicated that Weight growth velocity of EAT ≤4 days group (a*β*: -3.68, 95%*CI*:-5.12 ~ − 2.24) and EAT > 4 days group (a*β*:-4.83, 95%*CI*: − 6.12 ~ − 3.53) was lower compared to that of the non-antibiotic group. Weight GV of EAT > 4 days group was lower than that in EAT ≤4 days group (a*β*:-1.15, 95%*CI*:-1.96 ~ − 0.28). Days of reach of full enteral feeding in EAT > 4 days group was longer than that in non-antibiotic group (a*β*: 2.77, 95% *CI*: 0.25 ~ 5.87). The risk of feeding intolerance (FI) in EAT > 4 days group was 4 times higher than that in non-antibiotic group (a*OR*: 4.14, 95%*CI*: 1.49 ~ 13.56) and 1.8 times higher than that in EAT ≤4 days group (a*OR*: 1.82, 95%*CI*:1.08 ~ 3.17). There was no significant difference in the duration of parenteral nutrition, the age of oral calorie attainment and the incidence of EUGR among the three groups (all *P* > 0.05) (Table 3).

**Table 3:** Associations between empirical antibiotics treatment and nutritional outcome in VPIs

Outcomes	Antibiotic exposure			a*β*/*OR*^a^(95% *CI*) ≤4d vs 0d^b^	a*β*/*OR*^a^(95% *CI*) > 4d vs 0d^b^	a*β*/*OR*^a^(95% *CI)* > 4d vs ≤4d^c^
	non-antibiotic(*n* = 152)	EAT ≤4 days (*n* = 374)	EAT > 4 days (*n* = 1308)			
Weight growth velocity	16.8 (12.7,22.4)	14.6 (12.3,16.9)	14.3 (12.2,16.5)	-3.68 (−5.12 ~ −2.24)	-4.83 (−6.12 ~ − 3.53)	-1.15 (− 1.96 ~ − 0.28)
Days of reach of full enteral feeding	17.7 (12.0,26.0)	21.3 (15.4,28.1)	23.5 (16.6,33.2)	−0.39 (− 4.78 ~ 3.92)	2.77 (0.25 ~ 5.87)	2.46 (− 1.68 ~ 6.32)
Duration of parenteral nutrition	13.5 (10.0,20.0)	18.5 (12.5, 24.5)	19.0 (12.0,28.0)	−5.45 (−10.98 ~ 0.08)	−3.54 (−8.78 ~ 1.49)	2.15 (− 1.32 ~ 5.56)
Age of oral calorie attainment	17.5 (12.0,23.6)	20 (13.0,28.0)	21.0 (15.0,30.0)	−1.14(−4.69 ~ 2.41)	1.93(− 1.20 ~ 5.06)	3.08 (0.96 ~ 5.19)
FI	36 (23.7)	92 (24.6)	439 (33.6)	2.32 (0.55 ~ 8.62)	4.14 (1.49 ~ 13.56)	1.82 (1.08 ~ 3.17)
EUGR	50 (32.9)	159 (42.5)	592 (45.3)	0.95 (0.35 ~ 2.60)	1.25 (0.50 ~ 3.08)	1.35 (0.74 ~ 2.53)

^a^ Adjusted for GA, BW, antenatal corticosteroids, 5-min Apgar score, SGA, RDS, time to start enteral feeding, breast-feeding, duration of mechanical ventilation, cumulative fasting days, use of breast milk fortifier, anemia requiring blood transfusion

^b^ Reference is non-antibiotic group

^c^ Reference is EAT ≤4 days group

*FI* feeding intolerance, *EUGR* extrauterine growth retardation

### Effect of empirical antibiotics treatment on clinical outcomes

Univariate analysis revealed significant differences in the incidence of NEC ≥ stage 2, HAI, hsPDA, moderate and severe BPD and parenteral nutrition associated cholestasis (PNAC) (all *P* < 0.05) among the three groups (Table 4). After adjusting for factors that may affect the clinical outcomes, multivariate analysis showed that the risk of NEC ≥ stage 2 in EAT > 4 days group was 7.7 times higher than that of the non-antibiotic group (a*OR*: 7.68, 95%*CI*: 1.14 ~ 54.75) and 5.4 times higher than that in the EAT ≤4 days group (a*OR*: 5.42, 95%*CI*: 1.94 ~ 14.80). The risk of hsPDA incidence in EAT > 4 days group was 3.3 times higher than that of the non-antibiotic group (a*OR*: 3.28, 95%*CI*: 1.48 ~ 9.03) and 2.8 times higher than that in EAT ≤4 days group (a*OR*: 2.75, 95%*CI*: 1.54 ~ 4.88) (Table 5).

**Table 4:** Univariate analysis of the clinical outcomes of VPIs among the three groups

Outcomes	non-antibiotic	EAT ≤4 days	EAT > 4 days	*χ*^*2*^	*P*
	(*n* = 152)	(*n* = 374)	(*n* = 1308)		
NEC ≥ stage2,* n(%)*	1 (0.7)	6 (1.6)	85 (6.5)^a,b^	21.229	<0.001
HAI,* n(%)*	11 (7.2)	42 (11.2)^a^	180 (13.8)^a^	6.148	0.046
hsPDA,* n(%)*	6 (3.9)	24 (6.4)	219 (16.7)^a,b^	39.527	<0.001
IVH (grade 3 or 4),* n(%)*	1 (0.7)	3 (0.8)	22 (1.7)	2.295	0.317
PVL,* n(%)*	2 (1.3)	13 (3.5)	38 (2.9)	2.108	0.349
Moderate and severe BPD,* n(%)*	3 (2.0)	30 (8.0)^a^	147 (11.2)^a^	14.913	0.001
PNAC,* n(%)*	4 (2.6)	15 (4.0)	112 (8.6)^a,b^	14.171	0.001
ROP requiring intervention,* n(%)*	2 (1.3)	3 (0.8)	34 (2.6)	5.037	0.081

^a^ Significantly different between the non-antibiotic group and EAT group

^b^ Significantly different between the EAT ≤4 days group and EAT >4 days group

*NEC* necrotizing enterocolitis, *HAI* hospital-acquired infections, *hsPDA* hemodynamically significant patent ductus arteriosus, *IVH* intraventricular hemorrhage, *PVL* periventricular leukomalacia, *BPD* bronchopulmonary dysplasia, *PNAC* parenteral nutrition associated cholestasis, *ROP* retinopathy of prematurity

**Table 5:** Association**s** between empirical antibiotics treatment and clinical outcome in VPIs

Outcomes	Antibiotic exposure			a*OR*^a^(95% *CI*) ≤4d vs 0d^b^	a*OR*^a^(95% *CI*) > 4d vs 0d^b^	a*OR*^a^(95% *CI)* > 4d vs ≤4d^c^
	non-antibiotic(*n* = 152)	EAT ≤4 days (*n* = 374)	EAT > 4 days (*n* = 1308)			
NEC ≥ stage2	1 (0.7)	6 (1.6)	85 (6.5)	1.45 (0.17 ~ 12.82)	7.68 (1.14 ~ 54.75)	5.42 (1.94 ~ 14.80)
HAI	11 (7.2)	42 (11.2)	180 (13.8)	0.96 (0.71 ~ 1.28)	1.32 (0.85 ~ 1.47)	1.28 (0.78 ~ 1.43)
hsPDA	6 (3.9)	24 (6.4)	219 (16.7)	1.25 (0.42 ~ 3.56)	3.28 (1.48 ~ 9.03)	2.75 (1.54 ~ 4.88)
IVH (grade 3 or 4)	1 (0.7)	3 (0.8)	22 (1.7)	0.70 (0.06 ~ 7.15)	0.85 (0.14 ~ 6.44)	1.35 (0.46 ~ 4.25)
PVL	2 (1.3)	13 (3.5)	38 (2.9)	2.65 (0.59 ~ 12.05)	1.48 (0.33 ~ 6.50)	0.62 (0.42 ~ 1.26)
Moderate and severe BPD	3 (2.0)	30 (8.0)	147 (11.2)	3.75 (0.74 ~ 19.05)	3.24 (0.77 ~ 13.28)	0.85 (0.48 ~ 2.08)
PNAC	4 (2.6)	15 (4.0)	112 (8.6)	1.23 (0.65 ~ 3.44)	2.25 (0.78 ~ 8.34)	1.45 (0.93 ~ 1.77)
ROP requiring intervention	2 (1.3)	3 (0.8)	34 (2.6)	0.24 (0.02 ~ 3.61)	0.62 (0.06 ~ 5.85)	2.43 (0.47 ~ 10.34)

^a^Adjusted for GA, BW, sex, mode of delivery, antenatal corticosteroids, PROM> 18 hours, 5-min Apgar score, RDS, breast-feeding, duration of mechanical ventilation

*NEC* necrotizing enterocolitis, *HAI* hospital-acquired infections, *hsPDA* hemodynamically significant patent ductus arteriosus, *IVH* intraventricular hemorrhage, *PVL* periventricular leukomalacia, *BPD* bronchopulmonary dysplasia, *PNAC* parenteral nutrition associated cholestasis, *ROP* retinopathy of prematurity

## Discussion

Antibiotics are among the most commonly prescribed drugs in the NICU, despite absence of infection in most cases [22]. In the early stage of the disease or when the culture results are not available, up to 94% of antibiotics usage in the NICU is via EAT [22] and long-term use of antibiotics often appears in clinical practice. However, in VPIs, those clinical manifestations such as respiratory and circulatory instability, increased heart rate, feeding intolerance (FI), or body temperature fluctuation are not reliable indicators of infection [23]. Additionally, application of non-specific inflammatory indexes, such as C-reactive protein (CRP), procalcitonin, white blood cell count, or platelet count to determine duration of antibiotics use is questionable [24]. It is more recognized that continuous normal values of biomarkers like CRP and procalcitonin over the first 48 hours of age can help to eliminate infection and shorten the course of antibiotics [25].

The rate of antibiotics use in hospitalized premature infants remains high. An investigation from 24 tertiary medical institutions indicated that the average ratio of antibiotic use to hospitalization was 53.0%, with a maximum of 91.4% among VLBWI and ELBWI during hospitalization in China [26]. In a retrospective study from 297 academic and community hospitals across the United States [3], the majority of premature infants had early antibiotic initiation (31,715 VLBW infants [78.6%] and 11,264 ELBW infants [87.0%]). Collectively, a total of 11,669 cases (84.9%) of VLBWI from grade III NICU in Canada received EAT during hospitalization between 2010 and 2014 [6]. In our study, high frequency use of antibiotics (91.7% of EAT in VPIs without infection-related morbidities, even reaching 100% in some hospitals) and long course antibiotic use were also been found; 71.3% of EAT was used for more than 4 days, and the median period was 9 days, which exceed that of USA by more than 5 days [27] and that of Norway by more than 4 days [5]. Although there remains controversy on antibiotics duration when the cultures are negative, a 48-hour course with negative culture is sufficient for rule-out sepsis and will result in dramatic reduction of antibiotic use [25]. Moreover, a lower BW and GA, longer mechanical ventilation time, Apgar score ≤ 7 at 5 minutes, or postnatal corticosteroid use were found to be associated with increased EAT use and prolonged EAT. The high rate of EAT in newborns may be due to the following reasons: (I) Physicians believe that premature infants or invasive procedures may be associated with bacterial infection; (II) Postnatal corticosteroid immunosuppression may cause infection according to the belief of Chinese doctors, but there is no evidence-based basis for this. It is worth recommending that several recent studies used delivery characteristics to identify premature infants at lower risk of EOS. Puopolo and Mukhopadhyay conducted a study of 15,433 infants born at 22 to 28 weeks’ gestation in Neonatal Research Network centers from 2006 to 2014, those born by cesarean delivery with membrane rupture at delivery and absence of clinical chorioamnionitis were significantly less likely to have confirmed EOS [2]. Additionally, the neonatal EOS calculator is a clinical risk stratification tool increasingly used to guide the use of empirical antibiotics for newborns [28].

In recent years, numerous studies have demonstrated that unnecessary or long-term use of antibiotics can increase the risk of adverse clinical outcomes in premature infants, and prolonged use of broad-spectrum antibiotics can cause strong selective pressure on microorganisms, which will in turn induce drug-resistance [29]. In this study, a significant positive correlation was found between the risk of NEC ≥ stage 2 and EAT usage or prolonged EAT. Antibiotic-induced gut microbiota dysbiosis in preterm infants have been linked to the pathogenesis of NEC, which is consistent with our findings [30]. A large retrospective cohort study of 4039 extremely low birth weight infants by Cotten et al. [31], found that prolonged EAT (≥5 days) tended to be associated with NEC or death and NEC alone, with a ~ 4% increase in the odds of NEC or dying and a ~ 7% increase in the odds of NEC alone for each additional day of initial EAT. A meta-analysis of 13 studies involving 7901 premature infants, reported that the initial EAT ≥5 days correlated with the risk of NEC [32]. Similar associations were observed for hsPDA in our study. It has been reported that gentamicin, tobramycin, and other aminoglycoside antibiotics can relax arterial smooth muscle and delay the closure of PDA, with an increased risk of hsPDA [33]. However, no aminoglycoside antibiotics were used in our case. There is a paucity of biological data explaining the mechanism between antibiotic exposure and hsPDA, and whether other antibiotics have this adverse effect needs further study.

Moreover, clinicians tended to suspect infections when VPIs showed increased heart rate, blood pressure fluctuation and shortness of breath before the diagnosis of hsPDA, which resulted an increase in EAT.

Our study suggests that early empiric antibiotic exposure may result in decreased weight growth velocity, increased duration of reach of full enteral feeding, and higher risk of feeding intolerance among VPIs. There is a growing literature on the deleterious impact of antibiotics on neonatal nutritional outcomes. Martinez et al.found that early empiric antibiotic use was associated with delayed feeding tolerance in premature infants [34]. The gut microbiota of preterm infants influence nutrient absorption and this may be disrupted following antibiotics use [35]. An immature ontogenesis of the enteric microbiota may affect the pathophysiology of feeding intolerance [36]. Although there is no difference in the incidence of EUGR at 36 weeks of GA, our study points out that prolonged EAT has adverse effects on nutrition management of VPIs in a short-term process, which is not conducive to the nutritional development of VPIs during this period and may may contribute to NEC. On the basis of these results, we recommend that EAT should be avoided where possible, and the duration of antibiotic use should be shortened to reduce the incidence of adverse nutritional outcomes in premature infants, which can potentially reduce time of hospital stay and may save health-care resources.

This study had several limitations. Firstly, some indicators such as chorioamnionitis during pregnancy, antibiotics during pregnancy, cesarean section with no labor onset, neonatal umbilical vascular catheterization, central venous catheterization, and pulmonary surfactant use were not considered because of too many missing values. These confounding factors may have influenced the clinical outcomes. However, after adjusting for known confounding factors, the conclusion that prolonged use of EAT during hospitalization was associated with adverse effects on the clinical outcomes and nutrition management of VPIs did not change. Secondly, the data presented here come from a prospective multicenter study on factors influencing VPIs-EUGR in China, which excluded patients who died during hospitalization. Therefore, the effect of EAT on death as a clinical outcome was not studied. Thirdly, there is no equipoise at neonatal pediatrics treatment level and the policies of care in hospitals in China, which may also affect the short-term clinical outcome of newborns.

## Conclusions

In this study, we found that the utilization rate of EAT starting within 1 week after birth among VPIs was high despite absence of infection, particularly in younger gestational age and lower birth weight groups. Prolonged EAT within the first postnatal week was associated with decreased weight growth velocity, longer duration of reach of full enteral feeding, increased risk of feeding intolerance and NEC ≥ stage 2 after the first postnatal week. Therefore, stronger policies regarding the initiation and continuation of EAT among VPIs should be created to reduce the frequency and duration of EAT in Chinese NICUs.

## Data Availability

The datasets generated and/or analysed during the current study are not publicly available but are available from the corresponding author on reasonable request. Due to the data were used under license for the current study, and so are not publicly available.

## Abbreviations

EAT: Empirical antibiotics treatment
VPIs: Very preterm infants
FI: Feeding intolerance
NEC: Necrotizing enterocolitis
HAI: Hospital-acquired infections
hsPDA: hemodynamically significant patent ductus arteriosus
NICU: Neonatal intensive care unit
VLBWI: Very low birth weight infants
ELBWI: Extremely low birth weight infants
BPD: Bronchopulmonary dysplasia
LOS: Late-onset sepsis
EUGR: Extrauterine growth retardation
GA: Gestational age
BW: Birth weight
EOS: Early-onset sepsis
CSF: Cerebrospinal fluid
RDS: Respiratory distress syndrome
FI: Feeding intolerance
IVH: Intraventricular hemorrhage
PVL: Periventricular leukomalacia
PNAC: Parenteral nutrition associated cholestasis
ROP: Retinopathy of prematurity
SGA: Gestational age
GDM: Gestational diabetes mellitus
CDC: Centers for Disease Control and Prevention
